# Editorial: Protein Quality Controlling Systems in Plant Responses to Environmental Stresses

**DOI:** 10.3389/fpls.2018.00908

**Published:** 2018-06-29

**Authors:** Minghui Lu, Hanjo A. Hellmann, Yule Liu, Wei Wang

**Affiliations:** ^1^College of Horticulture, Northwest A&F University, Shaanxi, China; ^2^School of Biological Sciences, Washington State University, Pullman, WA, United States; ^3^MOE Key Laboratory of Bioinformatics, Center for Plant Biology, Tsinghua-Peking Joint Center for Life Sciences, School of Life Sciences, Tsinghua University, Beijing, China; ^4^College of Life Sciences, State Key Lab of Wheat & Maize Crop Science, Henan Agricultural University, Zhengzhou, China

**Keywords:** plant, heat shock proteins, unfolded protein response, proteasome, autophagy, abiotic stress

In nature, plants are routinely exposed to adverse environmental conditions, such as elevated temperature, drought, salinity, heavy metal, etc., which are among the main causes for declining crop productivity worldwide and lead to billions of dollars of annual losses (Dhankher and Foyer, 2018). These stressors negatively affect plant growth and development by inducing misfolding, denaturation, oxidation and aggregation of proteins. Evolutionally, plants have developed a comprehensive protein quality controlling system (PQCS) to maintain protein homeostasis, mainly including heat shock proteins (HSPs), unfolded protein response (UPR), ubiquitin-proteasome system (UPS) and autophagy. This research topic aims to summarize and report novel findings on the identification, functional analysis, signal transduction, transcriptional and post-transcriptional regulation, and protein interaction of candidate components in the above systems.

HSPs are abundantly expressed under abiotic stress conditions, and function as molecular chaperones to promote proper protein folding and to prevent denatured proteins from self-aggregation (Reddy et al., 2016). Yu et al. identified 42 putative *SlHSP20* genes from tomato (*Solanum lycopersicum*), and found that their transcript levels were profusely induced by abiotic stresses such as heat, drought, salt, but also by the fungal pathogens *Botrytis cinerea*, and tomato spotted wilt virus (TSWV). In addition, a total of 76 putative *CaDnaJ*/*HSP40* genes were identified in pepper (*Capsicum annuum* L.), and more than 80% of them responded to heat stress treatment (Fan et al.). These studies underscore the potential involvements of *HSP* genes in mediating the response of plants not only to elevated temperatures but also to a broader range of environmental stress conditions.

Endoplasmic reticulum (ER) is the major organelle for folding and assembling of secretory proteins. When plants are subjected to environmental stresses, the unfolded or misfolded proteins accumulate in the ER which is referred to as ER stress (Schröder and Kaufman, 2005), and which further activates UPR to enhance the operation of the ER protein-folding machinery (Duwi Fanata et al., 2013). Bao and Howell summarized in this research topic the latest progresses in UPR. The authors discuss recent findings that this pathway is not only associated with abiotic stress response, but is also required during normal vegetative and reproductive development. In addition, it fulfills critical roles in plant immunity, affecting bacterial and viral infections. Evidence that the UPR in multicellular organisms acts in a tissue specific manner comes from Cho and Kanehara. The authors measured expression of the immunoglobulin-binding protein gene *BiP3*, a marker for ER-stress. This gene was strongly up-regulated after treatment with the ER-stress inducer tunicamycin (TM). Interestingly, *BiP3* expression in the plant was not uniformly increased but more tissue specific. For example, the mRNA abundance of *BiP3* was preferentially increased in the vascular tissues, and leaf hydathodes. In the root tip, high expression was specifically observed in the columella, and the epidermal cell layer of the elongation zone. These findings indicate that in response to TM, plants emphasize certain tissues and/or organs to maintain ER homeostasis.

When stressed protein repair or folding demands exceed the cellular capacities, protein degradation systems such as UPS and autophagy are activated to remove misfolded proteins (Liu and Howell, 2016). The UPS marks proteins for degradation by attaching polyubiquitin chains to target proteins, which in turn leads to their degradation via the 26S proteasome (Liu and Howell, 2016). But degradation of misfolded proteins is only one aspect of the UPS function. The pathway represents a central regulatory tool that affects most cellular processes in plants. For example, ICE1 (INDUCER OF CBF EXPRESSION 1) is involved in chilling and freezing tolerance by promoting expression of the *CBF3* (C-REPEAT-BINDING FACTOR 3) transcription factor, and other cold-responsive genes (Chinnusamy et al., 2003). However, after ICE1 facilitated a cold-shock response, it becomes ubiquitinated by the E3 ligase HOS1 (HIGH EXPRESSION OF OSMOTICALLY RESPONSIVE GENE 1) followed by proteasomal degradation (Dong et al., 2006).

In this research topic, Yuan et al. cloned *HbICE1* from rubber trees (*Hevea brasiliensis*) and showed that its overexpression in *Arabidopsis* enhances cold tolerance. Fan et al. reported the identification of MaSINA1 (SEVEN IN ABSENTIA1), an E3 ligase from banana (*Musa acuminata*), which interacts with MaICE1, and promotes its degradation. Consequently, the authors suggested that MaSINA1 functions as a negative regulator of cold stress response in banana.

While UPS is targeting single proteins for degradation, autophagy is active on a broader scale and responsible for the degradation of single proteins, as well as protein aggregates or even whole organelles (Zientara-Rytter and Sirko, 2016). Autophagy is characterized by the *de novo* formation of a double membrane organelle (called autophagosome), to transport the targeted cargo components to the vacuole for degradation (Batoko et al., 2017). Autophagy-related proteins (ATGs) or their complexes recognize the target components by specific cargo receptors. In a review article, Wang et al. summarized the identification and functional characterization of three potential cargo receptors involved in plant abiotic stress, including NBR1 (NEIGHBOR OF BRCA1), TSPO (TRYPTOPHAN-RICH SENSORY PROTEIN), ATI1 (AUTOPHAGY INTERACTING PROTEIN1).

The timely degradation of misfolded proteins is important for the development of plant tolerance to abiotic stress. Luo et al. suggested that rapid protein turnover through autophagy is a prerequisite for the establishment of salt tolerance in *Arabidopsis*. They found that after salt treatment autophagosome formation is induced shortly, and the level of autophagy peaks within 30 min. Accordingly, within 3 h of salt treatment, accumulation of oxidized proteins is alleviated, and then contents of soluble sugar and some compatible solutes such as proline are enhanced. However, these processes are not observed or kept at lower levels in mutants such as *atg2* or *atg7* that are defective in autophagy. The authors propose that autophagy under salt stress is a critical requirement for bulk protein turnover.

The TOR (TARGET OF RAPAMYCIN) protein kinase is a major controller of growth-related processes in all eukaryotes. Under favorable conditions, TOR positively regulates cell and organ growth but restrains autophagy processes (John et al., 2011). However, Pu et al. reported that the modulation of autophagy by TOR was stress-type dependent. They found that the overexpression of the *TOR* kinase inhibited autophagy activation by nutrient starvation, salt and osmotic stress, but not by oxidative or ER stress. A similar result was observed after the treatment with the auxin NAA (1-naphthaleneacetic acid), a phytohormone that upregulates TOR activity. Since NAA treatment was unable to overcome blocked autophagy induced by a TOR inhibitor, it was suggested that auxin acts upstream of TOR in the regulation of autophagy.

Chen et al. found that KIN10 (KINASE HOMOLOG 10), a plant ortholog of the mammalian AMPK (AMP-ACTIVATED PROTEIN KINASE), acts as a positive regulator of autophagy by affecting the phosphorylation of ATG1 proteins in *Arabidopsis*. In *KIN10* overexpression lines (*KIN10*-OE), the stress-induced formation of autophagosomes were accelerated. In addition, leaf senescence was delayed, while the tolerance to nutrient starvation, drought and hypoxia treatments was increased. Furthermore, carbon starvation (transfer of seedlings to continuous darkness) enhanced the level of phosphorylated ATG1a in *KIN10*-OE lines.

Another aspect of autophagy in this research topic was investigated by Yan et al. by studying the impact of autophagy and D-glucose on the endocytosis of RGS1 (REGULATOR OF G-PROTEIN SIGNALING 1). Under normal conditions, RGS1 interacts with and arrests the GTPase activity of the heterotrimeric G-protein subunit Gα subunit (GPA1). However, D-glucose recruits WNK8 (WITH-NO-LYSING KINASE 8) to phosphorylate AtRGS1, which in turn causes its endocytosis. The endocytosis of RGS1 physically uncouples its inhibitory activity from GPA1, and then activates the G protein-mediated sugar signaling (Urano et al., 2012). Yan et al. reported that D-glucose induced RGS1 endocytosis is needed for the formation of autophagosomes likely by activating ATG8-phosphatidylethanolamine (PE) and ATG12/ATG5 conjugation systems. The autophagy pathway on the other hand is needed for RGS1 endocytosis as RGS1 remains associated with GPA1 in *atg2* and *atg5* autophagy mutants, even in the presence of D-glucose. The findings show a nice interplay between endocytotic and autophagy pathways, and shed new light on sugar signaling in plant cells.

The development of plant tolerance to abiotic stress always requires the simultaneous participation of different PQCSs. Heavy metals negatively affect plant cell viability mainly by disturbing protein folding and stimulating protein aggregation. In the review article of Hasan et al. the authors summarized the recent advances on the involvement of PQCSs in plant tolerance to heavy metal stress, including ion detoxification by phytochelatins and metallothioneins, reparation of damaged proteins by HSPs and UPR, degradation of denatured proteins by UPS and autophagy.

The proteomics study of Xu et al. provides us with new insights into the involvement of PQCS in establishing plant tolerance under adverse environmental conditions. Based on iTRAQ-quantitative proteomics approach, the authors compared the cucumber (*Cucumis sativus*) proteomes in adventitious roots under control and waterlogging conditions. They identified a total of 146 differentially regulated proteins (DRPs), of which 13 belonged to the categories of posttranslational modification, protein turnover and chaperones.

Polyamines such as putrescine (Put), spermidine (Spd) and spermine (Spm), are suggested to maintain the function and structure of cellular components in plant response to stress (Liu et al., 2015). After treatment with exogenous Put, Yuan et al. analyzed the DRPs of cucumber under salt stress by MALDI-TOF/TOF MS, and identified 62 DRPs, of which 15 functioned in protein metabolism, 15 in defense responses, 12 in carbohydrate metabolism, and 9 in amino acid metabolism. In a similar study in tomato with exogenous Spd, 67 DRPs were identified after high temperature treatment. The percentage of the identified proteins played roles in photosynthesis was 27%, followed by 24% of cell rescue, and defense. However, a significant amount was also related to protein synthesis, folding and degradation (22%) as well as energy and metabolism (13%) (Sang et al.).

The plant growth-promoting rhizobacterium (PGPR) can induce resistance against a broad spectrum of pathogens by simultaneously activating salicylic acid and jasmonate/ethylene-dependent signaling pathways (Niu et al., 2011). With a new potential strain NSY50, Du et al. investigated the mechanisms of PGPR protecting cucumber from the attack of *Fusarium oxysporum* f. sp. *Cucumerinum* (FOC) by a proteomic approach. Among the 56 DRPs, 14 belonged to the protein metabolism category and two to the HSP70 family, which suggests a functional connection between the PGPR and PQCS under biotic stress.

With the unprecedented global climate changes, extreme weather conditions are more likely to occur, and which will severely impact plant growth and crop production. A better understanding of the mechanisms of how plants are able to cope with and alleviate environmental stresses is essential for crop breeders to develop efficient strategies for maintaining our current agricultural productivity and to secure a sustainable agriculture. The research topic summarized here may provide some novel insights that can help to address these eminent challenges and to further increase crop production and secure yield in the upcoming decades.

## Author contributions

ML prepared the first draft of this editorial. HH, YL, and WW revised it. All authors listed approved it for publication.

### Conflict of interest statement

The authors declare that the research was conducted in the absence of any commercial or financial relationships that could be construed as a potential conflict of interest.
